# Play Badminton Forever: A Systematic Review of Health Benefits

**DOI:** 10.3390/ijerph19159077

**Published:** 2022-07-26

**Authors:** David Cabello-Manrique, Juan Angel Lorente, Rosario Padial-Ruz, Esther Puga-González

**Affiliations:** 1 Department of Physical and Sports Education, Faculty of Sports Sciences, University of Granada, 18071 Granada, Spain; dcabello@ugr.es (D.C.-M.); mrjuanangel11@correo.ugr.es (J.A.L.); mpuga@ugr.es (E.P.-G.); 2 Department of Didactics of Musical, Plastic and Corporal Expression, Faculty of Education Sciences, University of Granada, 18011 Granada, Spain

**Keywords:** badminton, health, benefits, systematic review

## Abstract

Regular physical activity (PA) engagement has multiple benefits for individual general health at all ages and life stages. The present work focuses on badminton, which is one of the most popular sports worldwide. The aim was to conduct a systematic review focused on examining and analysing this sport and the benefits it brings to the health of those who engage in it. Examination was conducted from the viewpoint of overall health and provides an overview of the current state-of-the-art as presented in published scientific literature. PRISMA 2020 guidelines were adhered to. An exhaustive search was conducted of four electronic databases or search engines: Web of Science, Scopus, MEDLINE and Google Scholar. The search terms used were “badminton AND health” and “badminton AND benefits”. In total, 27 studies were eligible for inclusion in the systematic review. After analysing the results, it was concluded that badminton engagement may lead to an improvement in all areas, the most studied being those related to physical health, in particular the improvement of cardiac and pulmonary functions and the development of basic physical capacities.

## 1. Introduction

The World Health Organization (WHO) establishes that health is the “state of complete physical, mental and social well-being and not merely the absence of disease or infirmity” [1]. This is currently considered to be framed by a set of determinants that include personal, biological, social, behavioural, economic, cultural and environmental factors which determine the state of health of individuals [2,3]. 

According to the WHO [1] definition, three types of health are established to make up comprehensive health:

Firstly, physical health refers to wellbeing of the body and optimal functioning of the organism. Secondly, mental health is considered as the absence of mental disorders or disabilities. It is a state of well-being in which individuals start to realise their capabilities and cope with the stresses of day-to-day life, work productively and contribute to their community. Thirdly, social health includes adaptation and self-management as skills used to face up to environmental changes and challenges, alongside the ability to establish satisfactory relationships with other people.

Regular physical activity (PA) engagement has been associated with important physical, mental, social and affective-emotional benefits. Specifically, the WHO [4] indicates that PA contributes to the prevention of non-communicable diseases, such as cardiovascular diseases, cancer and diabetes; reduces symptoms of depression and anxiety; improves reasoning, learning and judgement skills; ensures the healthy growth and development of young people and improves general well-being. Indeed, up to 5 million deaths a year could be avoided through greater PA engagement [4]. 

Physical inactivity is one of the most important risk factors for mortality (20–30% higher risk of death). According to WHO data (2019), one in four adults do not meet recommended PA levels. Issues around inactivity are currently heightened due to the pandemic (COVID-19) being experienced worldwide [5]. A study carried out by Wilke et al. [5] in a total of 14 countries showed that PA levels decreased during the pandemic.

The negative effects of inactivity have been widely studied, indicating poorer outcomes in academic performance [6]; mental health, such as higher levels of stress and anxiety [7,8,9,10,11]; physical health, associated with an increased risk of coronary heart disease [12,13]; reduced motor skill development [14,15] and lost opportunities to improve social relationships [16], among others.

Considering that participation in physical activity in general exerts benefits on well-being, health and social satisfaction [17], the present work sought to focus explicitly on badminton engagement and its impact on health. According to the International Federation of Sport for All [18] and the Madison Beach Volley Tour [19], it is also one of the most popular sports in the world, being widely practised worldwide, namely by more than 200 million individuals [20], since its inclusion in the 1992 Olympic Games [21]. This sport is characterised by high-intensity intermittent activity and has five events (men’s and women’s singles, men’s and women’s doubles, and mixed doubles), for which specific preparation is required in terms of technique, psychological control and physical fitness [21,22].

Scientific production in the field of badminton is scarce and diverse, focusing on the thematic areas of health and training [23,24] Although the number of publications has increased significantly since 2007, with Asian and European countries showing the highest rate of productivity [23,24], it is still low, particularly in terms of the benefits that this sport brings to the overall health of the athlete or player.

Despite the scarcity of studies, badminton, like other sports, has a number of health benefits. Recent studies provide significant effects of this sport on physical health, such as physiological improvements (increased power and high-density lipoprotein cholesterol and decreased blood pressure and resting heart rate) [25], the improvement of basic physical qualities [26] and improving the mental and social health of individuals [27,28].

For this reason, the present work is of great importance to the scientific community that has already extensively studied the most frequent injuries sustained in badminton, according to the competitive level of badminton practice, but never its benefits [29,30].

Thus, the present article reports a systematic review focused on the examination and analysis of the sport of badminton and the benefits it brings to the health of those who play it from the viewpoint of comprehensive health (physical, mental and social). Furthermore, analysis will be conducted as a function of age and sex. The aim of this is to provide an overview of the current state of the art.

## 2. Materials and Methods

The review was conducted in accordance with PRISMA guidelines laid out in “The PRISMA 2020: an updated guideline for reporting systematic reviews” [31].

### 2.1. Search Strategy

A comprehensive search was conducted of three electronic databases (Web of Science, Scopus and MEDLINE) between December 2020 and March 2021. A further update was then conducted in January 2022.

Given the scarcity of publications in this field, a number of databases were considered, and the search was not limited by the year of publication. Firstly, a more general search was carried out concering just “badminton” which produced many results (1693 in Scopus and 1581 in WoS), and a lot of information that did not fit the objective of our study: the benefits of practicing badminton. For this, we decided to refine the search to even more specific terms, such as mental health, but with a very low number of results; therefore, we decided to opt for the two expressions “health” and “benefits”, which were the ones that provided us with studies related to our objective, as other research has carried out with different sports, specifically regarding tennis [32].

Then, the final search terms used were “badminton AND health” and “badminton AND benefits”, also using the Boolean operator “and”.

### 2.2. Inclusion and Exclusion Criteria

Studies were eligible that (1) were scientific articles without exclusion of any type of research design; (2) were published in the English or Spanish language and had been peer reviewed; (3) examined badminton engagement with a view to attaining some type of comprehensive health benefit (cognitive, mental, physical, fitness, motor and social and emotional), regardless of age or the type of badminton engaged in.

In order to appropriately apply the presented inclusion criteria, the title of each identified paper and its abstract was first read. This was followed by a reading of the full text. Papers that did not examine the benefits of badminton were discarded.

The exclusion criteria applied were: (1) non-scientific articles, (2) articles published in languages other than English or Spanish, (3) articles not subject to peer reviewed and (4) articles that did not provide conclusions on the benefits of badminton practice in any of the areas of integral health.

### 2.3. Data Selection

All search results were exported to the Zotero library and duplicates were removed. The titles and abstracts of retrieved papers were screened by one reviewer, using the inclusion criteria described above and, subsequently, verified by another independent reviewer. If a study was mentioned several times, only the most recent publication was included in analysis. Reference lists of included studies, as well as related systematic reviews, were examined to identify any additional studies. The full texts of remaining papers were then reviewed to make a final decision on inclusion. Disagreements on the inclusion of studies were resolved by discussion with a third reviewer. Of the three experts, two were Masters in Sports Science and international badminton players and the third was a Ph.D. professor and badminton expert who is the Chair of the Sports Science and Medical Research Commission of the Badminton World Federation.

### 2.4. Data Extraction

Categorisation and analysis were performed using ATLAS.ti software (version 9). Data were extracted by one reviewer and checked for accuracy by another. The following characteristics were extracted and recorded for each included study:

Table 1: (1) papers; (2) authors; (3) year; (4) country in which study was conducted; (5) type of activity; (6) sample; (7) population and age; (8) intervention duration. Item (5), type of activity, referred to the type of badminton engaged in by participants and could include recreational, academic (referring to badminton played in an educational environment) and professional. Item (7) refers to the age of participants, which was classified, according to WHO criteria laid out on the Euroinnova website [33], as infancy (0–6 years), childhood (6–12 years), adolescence (12–20 years), youth (20–25 years), adulthood (25–60 years) and old age (60 upwards).

Table 2: (1) study design; (2) aim; (3) type of intervention program; (4) variables; (5) instruments; (6) health benefits of badminton.

Item (6), health benefits of badminton, referred to overall health, as stated in Section 2.2 of the inclusion criteria.

Study quality was analysed using descriptive statistics (absolute frequencies).

### 2.5. Assessment of Study Methodological Quality

The risk of bias in each eligible paper was assessed via a dichotomous nominal scale (yes/no), which was developed to assess sample adequacy in the 27 studies. Criteria used for continuous variables are listed in Section 2.2 (inclusion criteria). Inter-rater agreement pertaining to the classification of data gathered from included papers was 93%.

## 3. Results

### 3.1. Database Searches

**Figure 1:**
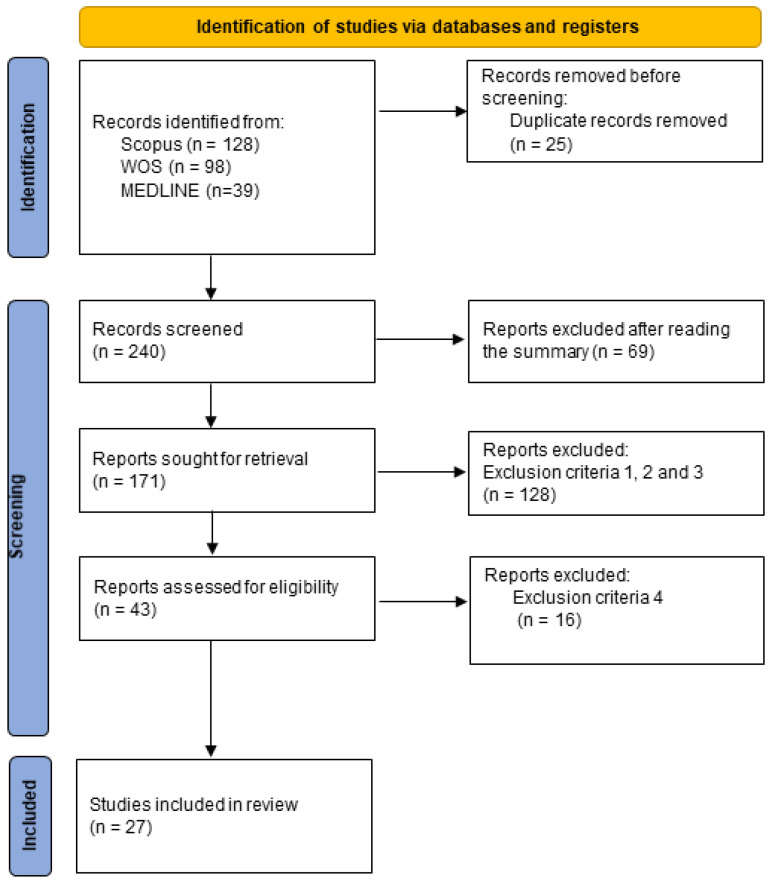
Research paper selection flowchart.

The PRISMA flowchart in Figure 1 illustrates the identification, selection, eligibility and inclusion of studies in the systematic review. The database search yielded 328 papers. In total, 27 studies were eligible for inclusion in the systematic review.

### 3.2. Description of Included Studies

Characteristics of included studies are described in Table 1 and Table 2

### 3.3. Findings Pertaining to the Characteristics of Selected Studies

With regards to the publication date of examined studies, an increase in the production of the literature on the subject can be seen in recent years, with 2020 being the most productive year, producing 25% of studies (n = 6), followed by 2019 (n = 4; 22.2%) and 2017 (n = 4; 14.8%). In terms of the countries in which studies were conducted, most studies were conducted in China and the United States (n = 4 in each country), followed by the United Kingdom, Turkey and Taiwan (n = 3 in each case).

In relation to the type of badminton considered by included studies, twelve papers were found on recreational badminton, eleven papers on academic badminton and four papers analysing professional badminton.

The total sample covered by the 27 included papers pertained to 20,983 participants. In terms of the sex of participants, 23 studies provide this information, corresponding to a total sample of 12,153 participants. Of these, 6308 men (51.9%) and 5845 (48.1%) women were considered.

When classifying papers according to population and age (Table 3), it was found that the population with which most studies were carried out pertained to adolescents (n = 11), with the least often examined population being children (n = 4).

The samples corresponding to the articles analysed refer to convenience samples in most cases (n = 22), either because they are expressly stated or because it is deduced after analysis of the text. Other articles used random samples (n = 4) and snowball sampling (n = 1).

Of the 27 papers analysed, the predominant study design used was experimental (n = 14). Of these, n = 8 were found to have used a control group, whilst n = 6 did not include a control group. Intervention durations ranged from less than 1 month (n = 3), 1 to 3 months (n = 6) and more than 3 months (n = 5).

The articles that carried out a badminton intervention programme (n = 15) had a variety of purposes, most of them related to the measurement of physiological parameters and fitness level or physical qualities (n = 12) and others to mental health (n = 3).

Examined variables were also diverse, with studies typically analysing more than one variable. The most commonly analysed variable was physical health (n = 17), followed by mental health (n = 10) and social health (n = 8) (Table 4).

With regards to data collection instruments, most studies used questionnaires (n = 13), with different physical condition tests (n = 6) and heart rate (n = 5) also standing out as being used to provide measures.

Finally, in terms of the results obtained, n = 15 articles reported significant positive improvements in several variables related to different types of health. Six articles found no significant differences in any of the study variables. No studies with negative significance were found.

## 4. Discussion

Through the practice of badminton, we can tackle physical inactivity, a worldwide problem that affects one in four people according to the WHO and, in turn, bring benefits to our overall health [4].

### 4.1. Physical Health Benefits

In consideration of physical health (improvement in physical and physiological parameters, physical and motor fitness and the absence of disease), three studies demonstrated benefits of badminton on cardiac function [25,45,49]. A study by Patterson et al. [43], examined adult women following eight weeks of badminton and showed a decrease in heart rate (HR) both at rest and during submaximal running. This finding was reiterated by research conducted by Chen et al. [28] and Ya and Li [49] with young men and women. These studies indicated that badminton was beneficial for cardiac function.

Several studies showing the benefits of badminton on respiratory capacity were also uncovered. In this sense, Patterson et al. [45] and Deka et al. [46] showed that badminton produced an increase in aerobic fitness and capacity (VO2max) in adults. Ya and Li [49] found the benefits of badminton on lung function in young men and women, whilst Dogruel et al. [43], in a study of children and adolescents of both sexes with asthma, showed that badminton decreased asthma symptoms and increased forced expiratory volume.

One study has also been conducted which demonstrates other benefits at a physical level. This study indicated a strengthening of the lens ligaments and normalisation of the ciliary muscle tone in boys and girls with different optical refractions following a one-year badminton engagement [40], whilst fewer postural asymmetries were found in adolescent boys playing badminton relative to adolescents not playing any sport [36]. Further outcomes included higher high-density lipoprotein (HDL) cholesterol levels, associated with a reduced likelihood of coronary heart disease, in adults and elderly men and women [39]; improved body shape in adolescent females due to the effect of badminton on development in the specific limb dimensions engaged during play [26] and better functional physical fitness and self-perceived functional health in the elderly, regardless of sex, alongside retarded biological degradation [54]. Higher bone mineral density in the femoral neck, humerus, lumbar spine and legs of male badminton players was also seen relative to those who played ice hockey or did not participate in any organised training activity [55]. Finally, Schnohr et al. [42], in a study carried out in young, adult and elderly people of both sexes, compared the life expectancy effects of engagement in various sports. These authors concluded that, relative to sedentary individuals, badminton players had a 6.2-year higher life expectancy, with this being the sport associated with the second greatest life expectancy benefit (tennis 9.7 years, badminton 6.2 years, football 4.7 years, cycling 3.7 years, swimming 3.4 years, etc.).

With regards to the benefits of badminton in terms of improving physical fitness, five studies reported benefits in adolescents of both sexes, such as improved muscular strength and endurance, explosive strength, power, flexibility, and cardiorespiratory fitness [26,34,35,73], obtaining significant improvements in all of the aforementioned parameters, with the only exception being body composition [35].

Yan and Li [49] also showed that badminton engagement in young people led to improved speed in both men and women, with better flexibility also emerging within women. In adults, Patterson et al. [45] showed improvements in vertical jump performance.

With regards to benefits at the motor level, Duncan et al. [37] conducted a study with children of both sexes and mainly focused on motor skills. They showed that both the quality and execution of motor skills improved following a BWF shuttle time structured program, with the most significant changes being obtained in younger children (6–7 years) rather than in older children (10–11 years). In addition, a significant gender difference was observed, with boys scoring significantly higher than girls on movement quality scores, regardless of age. Few studies were uncovered in young people and adolescents. In contrast, improvements in muscle coordination [50] and manipulative skills have been found in the elderly [54].

### 4.2. Mental Health Benefits

The present review identified badminton engagement to reduce depressive symptoms in young people with intellectual disabilities [25]. In adolescents, Zhao et al. [51] showed a decrease in depression and anxiety and improved self-esteem after 20 weeks of aerobic badminton exercise. In adult male and female patients with mental illness, Ng et al. [28] found that those who played badminton had greater overall motivation, one month after discharge, and improved psychological wellbeing [18].

At the cognitive level, five papers reporting benefits of badminton were uncovered. Takahashi and Grove [41] compared the effects of badminton on inhibitory function (the ability to control attention, behaviour, thoughts and/or emotions in order to overcome a strong internal bias or external attraction and instead do what is most appropriate or necessary). In Diamond [74], with results produced using simple running or sitting rest, as control conditions in young men and women, badminton significantly improved performance over sitting rest, whereas running did not. Similarly, a study conducted by Liao et al. [48] with male and female youth and adults, compared the effect of expertise on action inhibition in badminton players and non-athletes. Employing the stop-signal paradigm developed by Logan [68], this study found that badminton players were more likely to successfully inhibit their responses during stop trials than individuals who did not play sport, with response inhibition performance improving in line with the competitive level of badminton players. This underlines the relationship between cognitive ability and sport performance in badminton players.

Hung et al. [44] compared an open-skill exercise (badminton) with a closed-skill exercise (running) in young males, finding that badminton engagement resulted in higher levels of brain-derived neurotrophic factors and better task-switching performance, consequently improving executive function. In male adolescents and young adults, Dube et al. [50] demonstrated that badminton engagement resulted in a shorter visual reaction time compared to those who do not engage in any sporting activity, subsequently improving cognitive functions, alertness and concentration.

A study by Akin et al. [47] in children and adolescents of both sexes with autism spectrum disorder found that a 10-week badminton program improved attention.

### 4.3. Social Health Benefits

With regards to social benefits, Patterson et al. [45] found increased motivation to spend time with friends and establish new relationships amongst women. Through interviews with adults and elderly men and women, Chan and Lee [27] indicated that badminton was a conduit for self-expression and mood regulation, supporting personal development and social engagement [18]. Badminton also increased intrinsic motivation to perform tasks, the desire to compete (as a major benefit of participation) and general wellbeing [38]. In adolescents, badminton has been shown to increase motivation towards PA engagement [52].

The findings of the present review pertaining to the benefits of badminton engagement should be interpreted with caution and considered in light of the following limitations. Firstly, the high level of heterogeneity detected in the included studies (age, stage, study design, type of badminton played) limits the robustness of outcomes and reduces their generalisability. Secondly, due to the scarcity of studies conducted in this line of research, it is advisable to broaden the search to include papers published in more languages (such as Chinese, Korean, Japanese and French). This would be useful given that badminton is one of the most popular sports worldwide and it is highly likely that more research has been conducted in Asian countries. Finally, the disparity of the variables and instruments used to assess health improvement makes it difficult to compare the findings produced.

Although the study focuses exclusively on the benefits of practising badminton, without assessing other more negative or harmful aspects that the practice of any other sport always entails, such as the risk of injuries. However, the scientific literature already indicates that in the practice of amateur or recreational badminton, injuries are neither more numerous nor more important than those caused by the practice of any other sport or physical activity at these levels [29,30].

As a limitation of the study, the type of health and the variables within each of them, analysis is very diverse, with physical health being the most covered topic in the articles. A greater number of studies are needed in each of the areas of health described in this work, especially in mental and social health, in order to reach more reliable conclusions about the benefits of this sport.

## 5. Conclusions

As a general conclusion, it can be stated that the studies analysed demonstrate that badminton engagement can lead to all types of benefits associated with overall health improvement. Moreover, impact has been shown in all types of populations, ages and sexes. Furthermore, badminton, compared with other types of physical sporting activities, offers, for the most part, better outcomes pertaining to the three types of health (physical, mental and social), with benefits also seen for disabled individuals and even in visual health.

Conclusions pertaining to the specific benefits are presented in Table 5 for ease of understanding.

In conclusion, the present work provides coaches, monitors, practitioners, athletes and Physical Education teachers with specific guidance for carrying out badminton sports programs adapted to different populations and sexes with the aim of developing aspects of comprehensive health.

Despite the fact that in recent years there has been an increase in research on the sport of badminton, there is still a lack of studies on the health benefits it generates, so it is necessary to investigate in all areas but especially, given its current relevance, in mental and emotional health.

As future lines of research, following this review, we consider it of interest to focus research on the comparative analysis of the health effects between badminton and other types of sports and to reinforce studies on children and the elderly.

## Figures and Tables

**Table 1:** Main data gathered from selected studies.

#	Authors	Year	Country	Sport	Sample	Population and Age	Duration of Intervention
1	Lee et al. [34]	2021	South Korea	AB	120 high school students (65 M/55 W)	Teenagers. a:18.95	15 weeks per sport, three times a week, 35 min a session.
2	Chen et al. [25]	2021	USA	AB	18 participants with intellectual disabilities (14 M/4 W)	Young people. a:22.28	Ten lessons of 50 min, twice a week.
3	Lassandro et al. [17]	2021	Italy	PB	30 professional players	Adults. Age range of 18–69	-
4	Ng et al. [28]	2020	China	RB	84 patients (35 M/49 W)	Adults. a:36.7	-
5	Mohammed [35]	2020	Saudi Arabia	AB	346 M (FVC = 169; BC = 97; CG = 80)	Teenagers. (CG = a:19.61 ± 1.24 years; FVC= a:18.24 ± 0.56 years; BC = a:19.34 ± 0.68 years)	Twice a week for eight weeks, 50 min per session.
6	Chan and Lee [27]	2020	UK	PB	6 (4 M/2 W)	Adults and the elderly. a: 59.50 ± 6.37 years	-
7	Esen and Arslan [36]	2020	Turkey	AB	68 M (BA =14; BX = 12; JD = 14; TA = 14; NA = 15)	Teenagers. (BA = a:13.43 ± 0.94 years); BX = a:14 ± 1.71 years); JD = a:14.50 ± 1.95 years); TA = a:15.38 ± 0.96 years); NA = a:14.67 ± 0.49 years	-
8	Duncan et al. [37]	2020	UK	AB	124 children (67 M/57 W)	Children. m:8.5 ± 1.9 years	Six weeks, one session a week, 60 min per session
9	Buzzelli and Draper [38]	2020	USA	RB	3012 participants (1822 M/1190 W)	Elderly. m:63.17 years	-
10	Nassef et al. [39]	2019	Taiwan	RB	7797 people (3559 M/4238 W)	Adults and the elderly. 30–70 years	-
11	Tarutta et al. [40]	2019	Russia	RB	40 children with refractive errors of +6.63 a −6.75 D	Children. a:9.24 ± 1.06 years	6 months
12	Takahashi and Grove [41]	2019	Australia	RB	20 participants (8 M/12 W)	Young people. a:20.9 ± 0.2 years	3 days, ten minutes each day
13	Stovba et al. [26]	2019	Russia	AB	40 students W	Teenagers. Age range 17–18 years	2 times a week, 90 min per session.
14	Schnohr et al. [42]	2018	Denmark	RB	8577 (65% M, 35% W)	Young, adults and the elderly. Age range of 20–93 years. (BA = 44 ± 14 years).	-
15	Dogruel et al. [43]	2018	Turkey	AB	73 children (32 M/41 W) suffering from asm (SW = 27, SB = 26, ATL = 11, BA = 9)	Children and adolescents. a:12 ± 2.3 years	-
16	Hung et al. [44]	2018	Taiwan	RB	20 students W	Young people. a:23.15 ± 2.48 years	Two sessions of 30 min separated by a period of 7 days.
17	Patterson et al. [45]	2017	UK	RB	36 W healthy and untrained pre-menopausal	Adults. M:34.3 ± 6.9 years	8 weeks
18	Deka et al. [46]	2017	USA	RB	14 players W	Adults. M:35.9 ± 6.62 years	-
19	Akin et al. [47]	2017	Turkey	AB	3 girls with autistic spectrum disorder	Children and adolescents. a:12.6 ± 1.5 years	Adaptation exercises for two weeks and badminton exercises for 10 weeks.
20	Liao et al. [48]	2017	Taiwan	PB	42 badminton players (28 M/14 W) and 15 non-athletes (7 M/8 W)	Youth and adults. (BA = a:22.7 ± 1.5 years, NA = a:26.1 ± 2.6 years)	30 min
21	Yan and Li [49]	2015	China	AB	92 individuals of both sexes	Young people. M:20 years	15 weeks of training in five sports (basketball, badminton, qigong, aerobics and dancing)
22	Dube et al. [50]	2015	India	AB	100 M (50 belonging to the study group and 50 to the control group)	Teenagers and young people. Age range 18–22 years	-
23	Zhao et al. [51]	2014	China	AB	60 W non-athletic junior university students	Adolescents. a: 18.9 years	20 weeks, 4 days a week, 60 min a day
24	Stefanelli [52]	2014	Uruguay	RB	100 participants	Adolescents. Age range 12–13 years	9 months
25	Kim et al. [53]	2014	USA	RB	13 participants	Adolescents. Over 18 years old	
26	Lam et al. [54]	2011	China	RB	66 elderly individuals (27 M/39 W)	Elderly. Age range 65–75 years	10 weeks, two training sessions per week, 60 min per session
27	Tervo et al. [55]	2010	Sweden	PB	92 participants M (48 IH players, 19 BA players and 25 CP).	Teenagers and adults. Study outset: IH = 17 ± 0.4, BA players = 17.8 ± 2.5 and CP 16.9 ± 0.6. Study end: 29.0 ± 0.7 in IH players, 29.3 ± 3 in BA players and 29 ± 0.5 in CP players	-

Note 1 (Abbreviations). academic badminton (AB); recreational badminton (RB); professional badminton (PB); women (W); men (M); average (a); football and volleyball course (FVC); badminton course (BC); control group (CG); badminton (BA); boxing (BX); judo (JD); taekwondo (TA); non-athletes (NA); no exercise (NE); aerobic exercise (AE); swimming (SW); street basketball (SB); athletics (ATL); ice hockey (IH); control players (CP).

**Table 2:** Main data collected from analysed studies.

#	Design	Aim	Intervention	Variables	Instruments	Conclusion/Benefits
1	E (EG)	To investigate whether PA school programs (badminton and table tennis) affect health-related physical fitness.	Badminton and table tennis PA school program	Strength, power, cardiorespiratory fitness, flexibility, body mass index (BMI).	Dynamometer. Horizontal jump, shuttle race test, sit and reach test, stadiometer	Improved muscle strength, power, cardiorespiratory fitness (endurance), flexibility and total health-related fitness score.
2	E (EG/CG)	To assess the impact of badminton classes on the health and wellbeing of young adults with intellectual disabilities.	Introductory shuttle time badminton lessons	Resting heart rate (HR), blood pressure, circumference/waist, motor performance, motor skills, depression, self-confidence	Tensiometer (OMRON-BP742N), tape measure, emotiv COPD, 6-min walking test (PM6M), individual assessment of badminton skill, depression scale [56], short scale of self-efficacy [57].	There was a reduction in resting heart rate, an improvement in 6-min walk test performance and badminton skills. Increased frontal alpha asymmetry, which may have been due to decreased depressive symptoms in the exercise group.
3	L	To compare indicators of wellbeing and health perceptions between badminton master athletes and the adult population	-	Perception of physical and psychological health	Questionnaire on perceived quality of life, included within the Italian surveillance database “PASSI” of 2014–2017	Significantly better perceptions were detected in high-level athletes compared with the general Italian population. Badminton improved health, psychological wellbeing and social engagement.
4	L	To examine associations between severe mental illness, general health symptoms, mental wellbeing and activity levels.	-	Patients’ somatic and mental health	Brief psychiatric assessment scale (BPRS) [58], health questionnaire (PHQ-15) [59], Pittsburgh Sleep Quality Index (PSQI) [60], mental wellbeing scale (C-SWEMWBS) [61], motivation for general activity (GAMM) [62].	Engagement in badminton and tai chi was considered a predictor of motivation for general activity, one month after the end of the program.
5	E (2 EG/1CG)	To determine the effect of different Physical Education courses based on different sports or games on the health of university students.	Badminton training	Cardiovascular fitness, muscular endurance, explosive power, body composition, flexibility.	Cooper test, sit-ups, standing jump length, sit and stretch test	Significant improvements in all measured fitness parameters, except for body composition.
6	L	To explore the lived experiences of older people and the meaning of participation in sports, wellbeing and personality.	-	Wellbeing and personal development.	Semi-structured interviews	Improved personal development, self-expression and mood regulation. Sports participation supports wellbeing and continuous personal development in adulthood.
7	T	To check the postural differences between students who play sports (boxing, judo, taekwondo and badminton) and those who do not.	-	BMI, posture	Precision weighing instrument, B.A.K (body analysis capture)	Non-athletes had more postural asymmetries than athletes who engaged in sports such as badminton.
8	E. (EG/CG)	To examine the effects of a program on fundamental movement skills.	Structured shuttle time program	Motor skills, basic physical qualities.	Thick motor development test-2 (TGM-2) [63], Smart Speed Doors	Improvement in the quality and outcome of motor skills, with these being more significant in children aged 6–7 years.
9	T	To identify motivations for and perceived benefits of participating in pickleball in older adults.	-	Intrinsic and extrinsic motivations towards PA. Orientations towards success, perceived benefits.	Sports motivation scale. Task and ego orientation towards sport questionnaire, quality and importance of recreational services, developed by the National Intramural and Recreational Sports Association	Participants were more inclined towards the task than ego, more intrinsically motivated to perform such tasks and felt that engagement increased their desire for competition, general wellbeing and PA.
10	T	To compare high-density lipoprotein cholesterol levels between three groups of participants aged 30–70 years and classified according to physical exercise status: No exercise/aerobic/badminton.	-	Demographic, biochemical and lifestyle variables.	Data (demographic, biochemical and lifestyle) obtained from the Taiwan Biobank database.	Badminton engagement was associated with higher levels of high-density lipoprotein cholesterol. Higher HDL-C (high-density lipoprotein cholesterol) led to a lower risk of coronary heart disease.
11	E (EG)	To compare the level of aberrations, wavefront structure and its response to cycloplegia in children with different refraction profiles after a badminton program.	Badminton engagement.	Aberrations of the eye wavefront.	Aberrometer OPD-Scan III	Regular badminton engagement produced significant changes in wavefront aberrations, which is indicative of a strengthening of the ligaments of the lens and normalization of the tone of the ciliary muscle.
12	E (EG)	To compare the effects of badminton and running on inhibitory function.	Running, playing badminton and resting sitting down.	Aerobic capacity, heart rate, range of perceived exertion, volitional exhaustion, inhibitory function.	Motor treadmill, indirect calorimetry system (MetaMax-3B), Polar heart rate monitor (Model RS800cx), reverse Stroop task	A single session of complex exercise (badminton) produced greater benefits to inhibitory function than one session of simple exercise (running).
13	E (EG/CG)	To analyse the benefits of an academic physical education model, based on badminton, on the physical qualities and anthropometric characteristics of students.	Badminton-driven academic Physical Education model.	Physical qualities, anthropometric characteristics.	Push-ups in prone position, standing long jump, seated push-ups, 100 metre sprint test and 2000 metre race, circumference (size) and length measurements	The program improved speed by 10%, flexibility by 12%, endurance by 6% and power by 8%. It was also beneficial for body shaping, increasing the muscle mass of sport-specific limbs.
14	L	To examine the impact on life expectancy of participation in various sports.	-	PA levels, alcohol consumption, diabetes, blood pressure, cardiorespiratory fitness, strength, self-rated health, social network, vital exhaustion.	PA questionnaire, sphygmomanometer, electrocardiogram, blood tests	Badminton engagement increased life expectancy by 6.2 years when compared with a sedentary group.
15	L	To investigate the effect of regular exercise on asthma symptoms, quality of life and lung function in children with asthma.	-	Asthma symptoms, forced expiratory volume. Physical, mental and social disorders.	Asthma measurement form, Quality of life questionnaire in children with asthma (PAQLQ) [64], spirometer	Badminton engagement significantly improved asthma symptoms and increased forced expiratory volume.
16	E (EG)	To compare the effect of badminton engagement and running on brain-derived neurotrophic factor (BDNF) production and task change performance.	Badminton session	Cardiac frequency, perceived effort, haematocrit level, brain-derived neurotrophic factor, cognitive performance, reaction time.	Wireless heart rate monitor (BioHarness Team System), perceived effort scale, venous blood machine, task change paradigm [65].	Badminton led to higher levels of brain-derived neurotrophic factor compared with running. Demonstrated benefits of practising open skills (badminton) on brain-derived neurotrophic factor and executive function.
17	E (2EG/1CG)	To examine the effects of engaging in 8 weeks of recreational badminton in untrained women.	Badminton training	Physiological parameters, vertical jump, Height and body mass, body fat percentage, body density, exercise motivations, physical self-esteem.	Microvettes (CB300), Randox Monza UK analyser, Biosen C-Line Analyzer (EKF Diagnostics), sphygmomanometer (Omron M5), treadmill, Erymetro (Oxycon Pro), force platforms (PS2142), BODPOD scales, anthropometric tape (Lufkin W606 PM), reasons for exercise questionnaire (EMI-2; [66]). Physical self-perception.	Increased VO2max to exhaustion, vertical jump height, social commitment and motivation towards exercise. Decreased blood pressure and heart rate at rest and during submaximal running.
18	L	To determine oxygen consumption during an RB match and intensity, measured according to American College of Sports Medicine criteria and categorised as moderate or vigorous.	Badminton matches	Aerobic capacity, lactate concentration, range of perceived exertion, step count, heart rate, energy expenditure	Portable metabolic system, lactate analyser, Borg scale [67], pedometer, Polar watch and chest strap	RB was categorised as being of vigorous intensity, providing a potential means of meeting recommended PA levels and improving aerobic fitness.
19	E (EG)	To investigate the effect of simplified badminton exercises on the development of attention and retention level in individuals with autistic spectrum disorder.	Badminton Exercise Program	Level of care, mental retention capacity	Attention test, visual memory test, effect test.	A positive effect was achieved in the level of attention, development of eye contact, prolonged focusing on objects and attention development.
20	E (EG/CG)	To investigate the effect of expertise on action inhibition in badminton players and non-athletes.	Cognitive tests	Motor inhibition	Stop sign paradigm [68]	Badminton players were more likely to successfully inhibit their responses during stop trials, with responses being better in those who competed at higher levels.
21	E (EG)	To examine the effect of five sports on the physical health of students following fifteen weeks of training.	Special training in five different sports	Vital capacity, resistance, velocity, grip strength, squats, jumps, sit-ups	“China’s National Student Physical Health Standard” (CNSPFS).	Badminton had a beneficial effect on heart and lung function, speed and flexibility. In addition, it led to significant improvements in flexibility in girls.
22	T	To compare the visual reaction time of badminton players with control individuals of the same age.	-	Visual reaction time	Visual reaction time recorder	Badminton is beneficial in terms of improving hand-eye reaction time, muscle coordination, cognitive functions, alertness and concentration.
23	E (EG/CG)	To assess the effects of 20 weeks of aerobic exercise on symptoms of depression, anxiety and self-esteem in non-athlete university students.	Aerobic badminton exercise	Depression, anxiety, self-esteem	Depression inventory [69], inventory of state-trait anxiety [70], self-esteem inventory [71].	Students who performed badminton exercises significantly decreased levels of depression and anxiety and improved their self-esteem.
24	L	To examine whether badminton facilitates the “participation” of adolescents in other activities.	School year dedicated to badminton	Level of effective participation	Observation	Participation in badminton classes may be a strategic and determining factor of increasing and maintaining adherence to PE classes.
25	L	To examine the benefits of PA engagement in members of the same ethnic group.	-	Perceived benefits	Interview	Korean immigrant participants gained various social and cultural benefits. Highlight the psychological benefits perceived by participants.
26	E (EG/CG)	to investigate the effects of a combined Tai Chi and badminton training program on the functional physical health of older people.	Combined tai chi and badminton training	Functional physical health, strength, flexibility, agility, balance, aerobic resistance, hand–eye coordination	Activities of daily living scale (OARS-IADL) [72], physical fitness test for seniors, hand grip strength test, AAHPER fitness test	Functional physical health, manipulative skills and self-perceived functional health were improved. In addition, the program can serve as a tool to slow down inevitable biological degradation as individuals aged.
27	L	To investigate the influence of different types of PA using weights on bone mineral density and evaluate final residual benefits.	-	Bone mineral density, fatty acid profile, vitamin D, markers of bone metabolism.	Lunar DPX-L dual energy X-ray absorber, liquid chromatograph HP1100	Data indicated higher bone mineral density in the neck of the femur, humerus, lumbar spine and legs of badminton players relative to hockey players.

Note 1. Experimental design (E); Longitudinal (L); Transversal (T); Experimental group (EG); Control group (CG).

**Table 3:** Populations examined by included studies.

Population	Articles	Percentage
Children	n = 4	14.8%
Adolescents	n = 11	40.7%
Young people	n = 7	25.9%
Adults	n = 8	29.6%
Elderly	n = 5	18.5%

**Table 4:** Types of health examined.

Type of Health	Number of Articles	Percentage
Physical health	n =17	63%
Mental health	n =10	37%
Social health	n =8	29.6%

**Table 5:** Benefits produced by badminton engagement in different populations and sexes.

Type of Health	Specific Benefits	Population and Gender
**Physical Health**	Improvements in heart function	Adult women, young women and men, and young women and men with intellectual disabilities [25,45,49]
	Improvements in lung function (breathing capacity)	Adults of both sexes, young people of both sexes, children and adolescents of both sexes [43,44,45,46,49]
	Strengthens the ligaments of the lens	Children with various optical refractions [40]
	Reduces postural asymmetries between those who engage in and do not engage in sport.	Adolescents of both sexes [36]
	Lower risk of coronary heart disease	Adults and elderly men and women [39]
	Body shape benefits	Adolescent girls (16)
	Strengthens functional physical health and self-perceived functional health and slows biological degradation	Elderly men and women [54]
	Increases bone mineral density in the neck of the femur, humerus and lumbar spine	Adolescent males as they progress into adulthood [55]
	Increases life expectancy beyond other sports such as football, cycling and swimming.	Young, adult and elderly men and women [42]
	Improves basic physical abilities (speed, flexibility, endurance and strength)	Adolescents of both sexes [26,34,35]
	Improves speed	Young men and women [49]
	Improves flexibility	Young women [50]
	Improves vertical jump	Adult women [44]
	Better muscle coordination	Adolescents and young men [50]
	Improves manipulative skills	Elderly men and women [54]
	Improves motor skills	Boys and girls, with the most significant changes in the youngest children [37]
**Mental health**	Improves inhibition	Young people and adults of both sexes [41,48]
	Improves cognitive function, alertness and concentration	Male adolescents and young adults [50]
	Improves attention	Children and adolescents of both sexes with autistic spectrum disorder [47]
	Reduces depressive symptoms	Young men and women with intellectual disabilities and adolescent girls [25,51]
	Predictor of general motivation for activity	Adult patients of both sexes referred to occupational therapy [28] and adolescents [52]
**Social Health**	Improves social relationships	Adult women [45] and professional players [18]
	Supports personal development and mood regulation	Adults and elderly men and women [27]
	Increases intrinsic motivation	Elderly men and women [38]

Note: Numbers in brackets pertain to the reference of the paper from which the various benefits are drawn.
